# Supplementary material for: Underwater Leidenfrost nanochemistry for creation of size-tailored zinc peroxide cancer nanotherapeutics
Source: Nat Commun. 2017 May 12;8:15319. doi: 10.1038/ncomms15319 (PMC5437293; doi:10.1038/ncomms15319)
Supplement: Supplementary Information — Supplementary Figures. [file ncomms15319-s1.pdf]

Supplementary Figure 1.

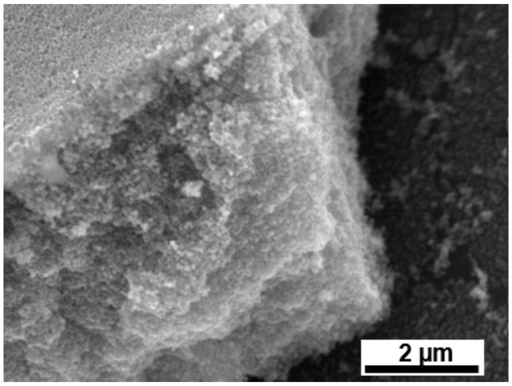

Supplementary Figure 1. SEM image of the synthesized monodisperse ZnO_2 nanoparticles by the beaker approach at a low magnification

Supplementary Figure 2.

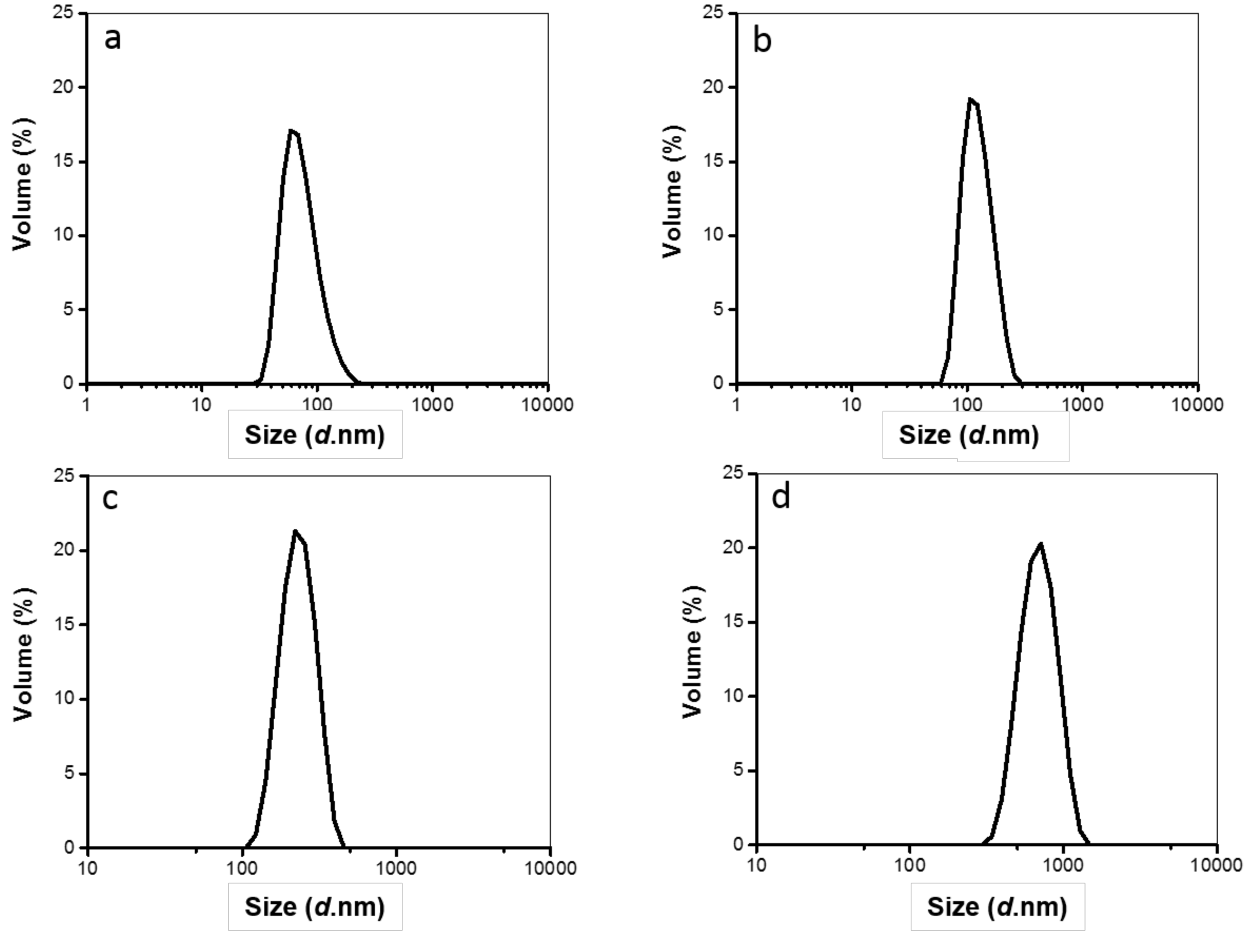

Supplementary Figure 2. Particle size distributions of ZnO_2 nanoparticles with the average size of a) 70 nm (poly dispersity index (PDI)=0.095); b)126 nm (PDI=0.03); c) 220 nm (PDI=0.005); d) 680 nm (PDI=0.12)(Low PDI implies monodispersity of the nanoparticles).

Supplementary Figure 3.

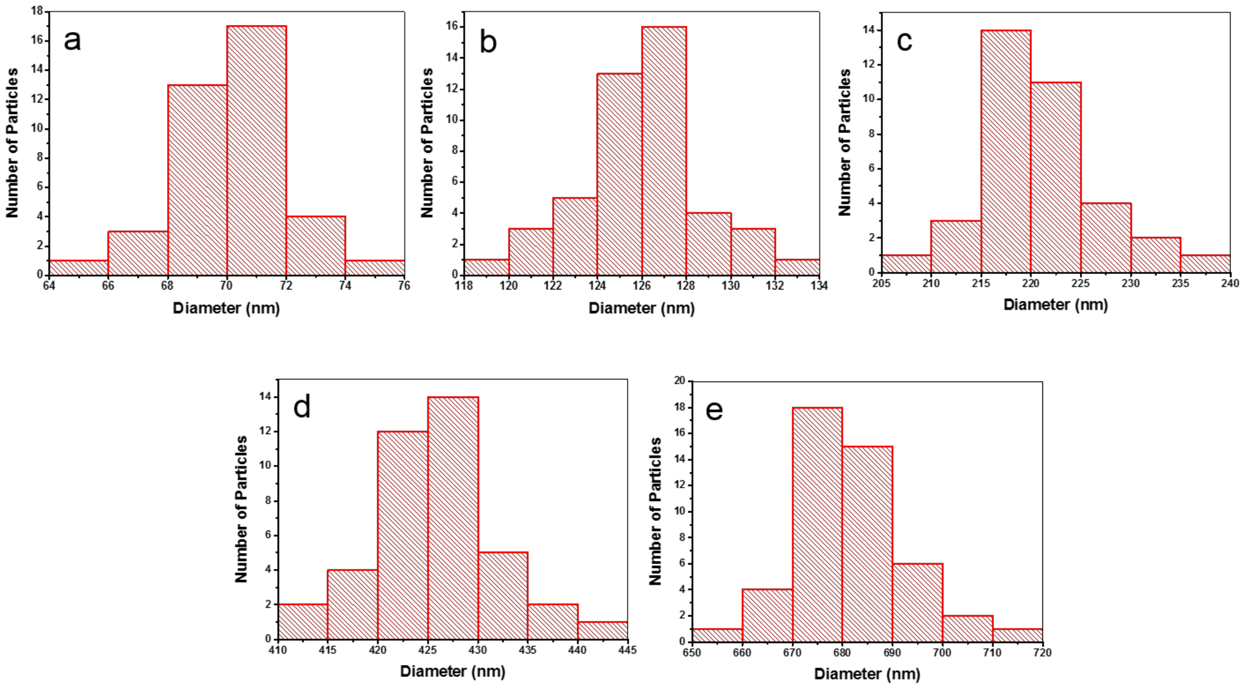

Supplementary Figure 3. Histograms of particle size distributions of ZnO_2 nanoparticles with the average size of a) 70 nm; b)126 nm; c) 220 nm; d) 426 nm; e) 680 nm (extracted from the SEM images labelled as Figure 2g,f,h,b and i, respectively).

Supplementary Figure 4.

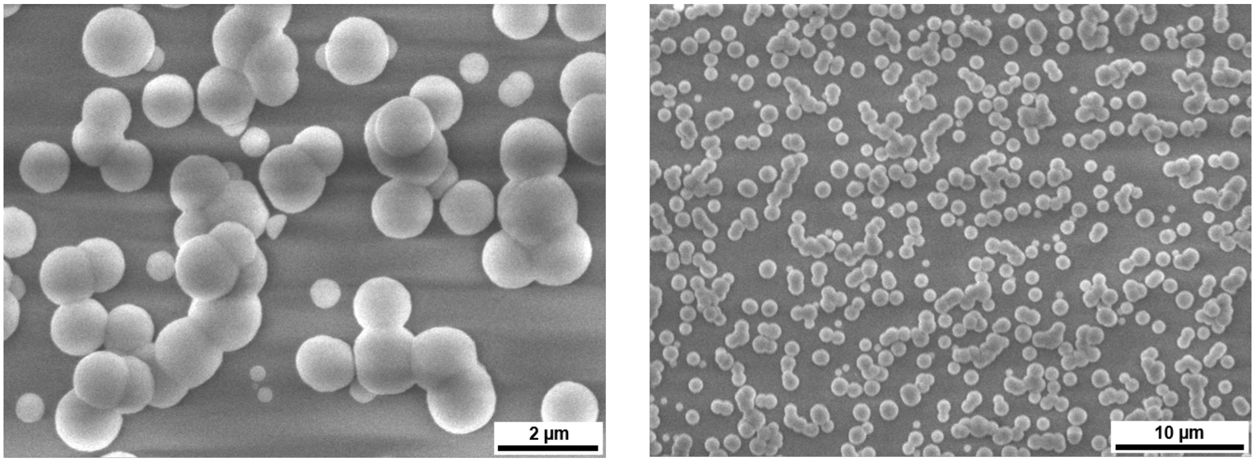

Supplementary Figure 4. SEM images of heterodisperse ZnO_2 nanoparticles synthesized by the classic Leidenfrost technique at two different magnifications. The nanoparticles were synthesized based on a precursor solution of 10 mM zinc acetate.

Supplementary Figure 5.

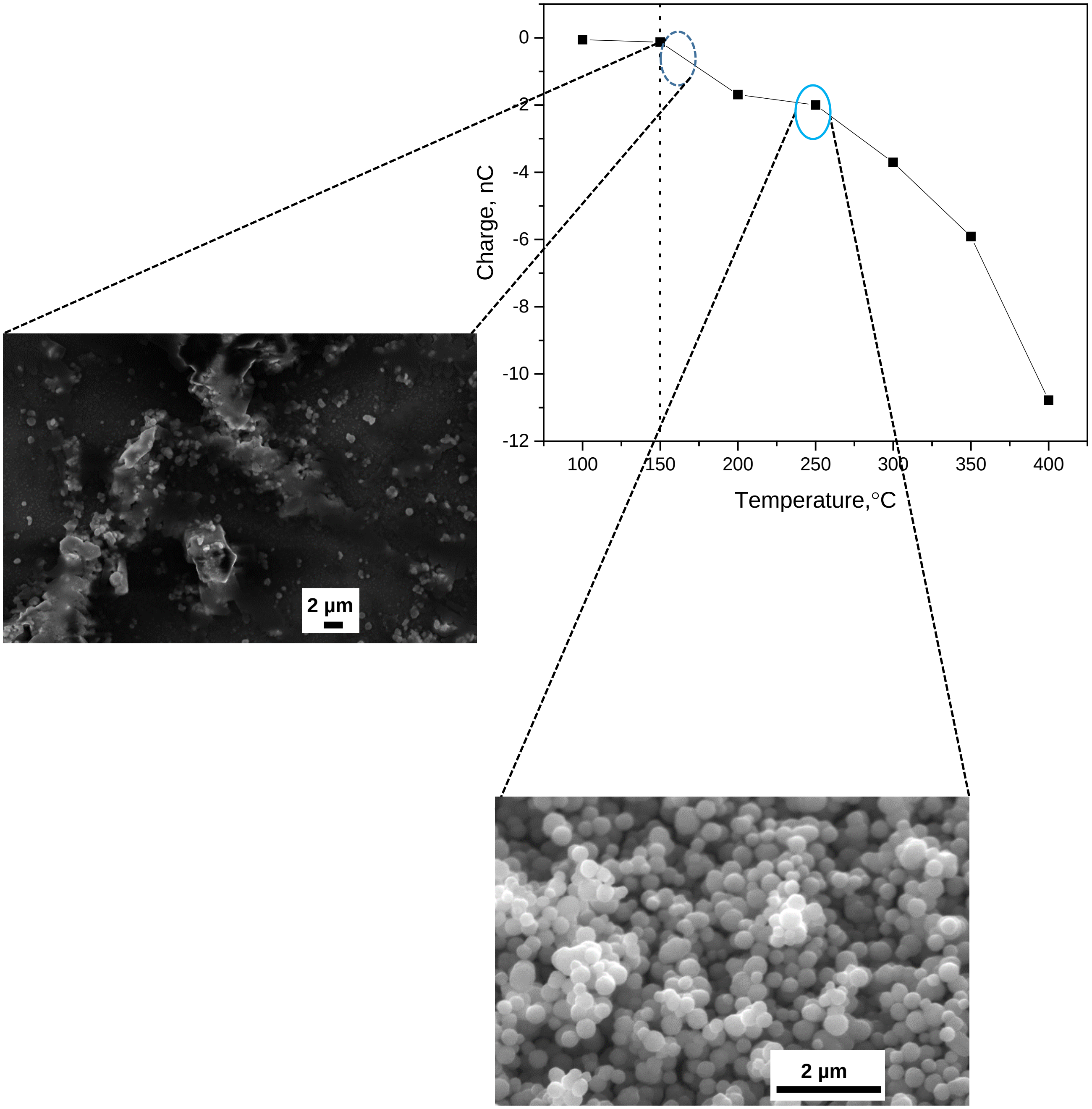

Supplementary Figure 5. Relationship between temperature of the hot plate thus water bath with the induced charge in the bath reactor (SEM images show the samples containing the major fraction of salt against particles and vice versa at the transition boiling state and at onset of the Leidenfrost state, respectively).

Supplementary Figure 6.

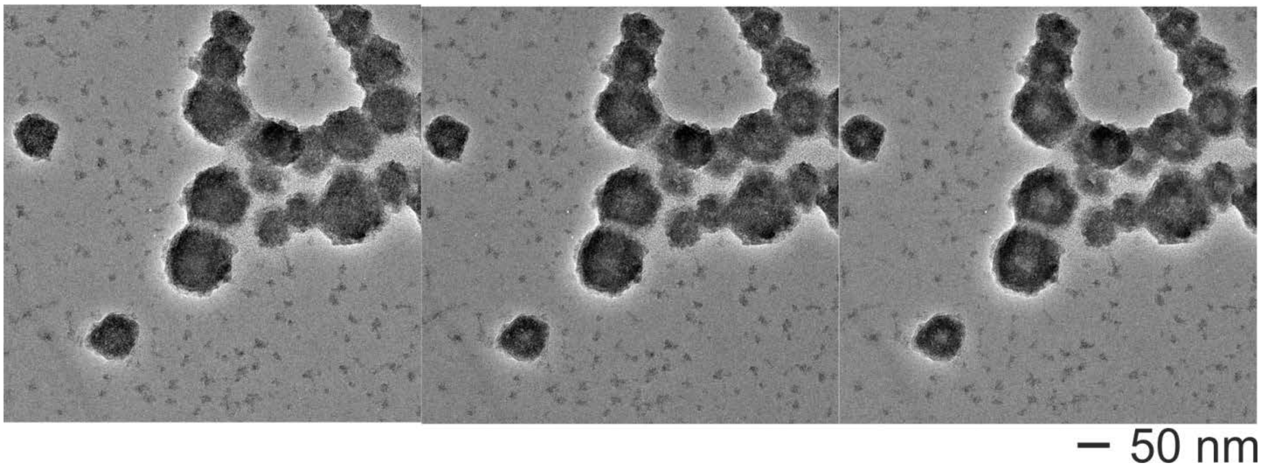

Supplementary Figure 6. Time resolved TEM images which show the formation of spheres due to electron beam irradiation

Supplementary Figure 7.

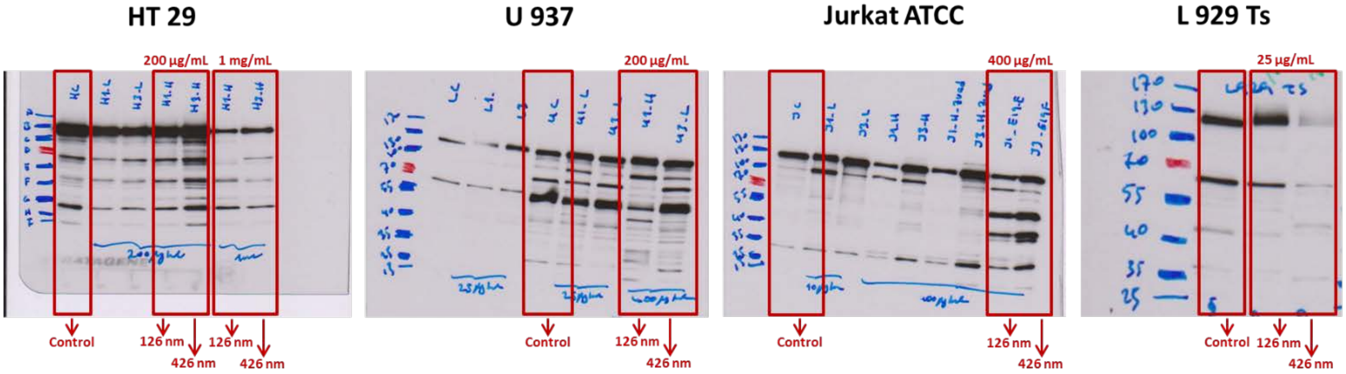

Supplementary Figure 7. Primary data for Figure 4g, i.e. the western blots of the studied cell lines, as camera images with visible molecular weight markers.
